# Ultrasound measurement of optic nerve sheath diameter pre- and post-lumbar puncture

**DOI:** 10.1186/s13089-020-00173-8

**Published:** 2020-05-13

**Authors:** Christopher K. Schott, Mohammad I. Hirzallah, Rock Heyman, Daniel N. Lesky, Emily B. Brant, Clifton W. Callaway

**Affiliations:** 1 VA Pittsburgh Health Care Systems * Critical Care Service Line (124U), University Drive, Pittsburgh, PA 15240 USA; 2 Department of Critical Care Medicine and Emergency Medicine, University of Pittsburgh, and University of Pittsburgh Medical Center (UPMC), 3550 Terrace Street Scaife Hall, Suite 600, Pittsburgh, PA 15213 USA; 3 University of Pittsburgh Multidisciplinary Critical Care Training Program, 3550 Terrace Street Scaife Hall, Suite 600, Pittsburgh, PA 15213 USA; 4 Department of Neurology, University of Pittsburgh, and University of Pittsburgh Medical Center (UPMC), 300 Halket Street, Suite 4500, Pittsburgh, PA USA 15213; 5 School of Medicine 401 Scaife Hall, University of Pittsburgh, 3550 Terrace Street, Pittsburgh, PA 15261 USA; 6 Department of Emergency Medicine, University of Pittsburgh, and University of Pittsburgh, Medical Center (UPMC), 3600 Forbes at Meyran Avenue Forbes Tower, Suite 10028, Pittsburgh, PA 15213 USA

**Keywords:** Optic nerve sheath diameter, Intracranial pressure, Ultrasound

## Abstract

**Background:**

To test the hypothesis that optic nerve sheath diameter (ONSD) correlates with real-time changes in intracranial pressure, we performed ultrasound measurements of the ONSD in ambulatory patients undergoing elective lumbar puncture (LP). We conducted a prospective cohort study, including adult patients undergoing LP in a non-emergent setting. We measured ONSD perpendicular to the optic nerve at 3 mm behind the globe in both eyes in the traverse and sagittal planes, with the patient supine. The primary outcome was change in ONSD from pre-LP to post-LP. We calculated association of opening and closing LP pressures with changes in the pre- and post-LP ONSD measurements.

**Results:**

The mean patient age was 49.0 years (SD = 37–61, range 19–67) with 21 females (72.4%) and 26 (89.7%) white American (not Hispanic or Latino). The average opening pressure and closing pressures were 20.4 cm and 13.5 cm with a difference of 6.9 cm, (95% CI 3.9–10.0 cm). Pressures between the participants with baseline ONSD measurement > 5 mm (average opening pressure = 21.3 cm) to those < 5 mm (20.2 cm) differed by 1.1 cm (95% CI − 5.7 to 8.0). Linear regression revealed no association between the sagittal, transverse, average, and change in ONSD measurements with the observed LP opening pressure, change in LP pressure, or volume of cerebral spinal fluid (CSF) drained.

**Conclusions:**

In this study of ambulatory patients undergoing rapid decreases in ICP via elective LP, we detected no acute changes in ultrasonographic measurement of the ONSD.

## Background

Optic nerve sheath diameter (ONSD) measurement by ultrasonography is a promising method to detect elevated intracranial pressure (ICP) [1, 2] and is gaining popularity as a beside assessment of ICP in critically ill patients [3]. This may be of particular benefit to undifferentiated patients presenting to the emergency department with concerns for elevated ICP and where Point-of-care ultrasound (POCUS) has a significant role in patient care.

Increased intracranial pressure transmits directly along the optic nerve and leads to papilledema [4, 5]. Several studies found that increase in ONSD correlates with increase in ICP [6–9]. Meta-analyses and systematic reviews support use of ONSD as a non-invasive ICP assessment modality [10]. However, different studies report variable inter-rater agreement and wide confidence intervals in measurements, suggesting caution-pending larger and more comprehensive studies [3, 11, 12].

In some studies, ICP and ONSD change simultaneously. These include when CSF drainage is used to reduce ICP [6, 13, 14], when ICP rises during endotracheal suctioning [15] and when intrathecal infusion tests increase ICP and are followed by intrathecal crystalloid injections that decrease ICP [16]. However, a cohort of subarachnoid hemorrhage patients showed that ONSD values did not normalize after normalization of ICP [17] and a study of relapse-free multiple sclerosis patients showed that the ONSD was smaller than normal controls [18].

This variability in ONSD changes by pathology and chronicity suggests that studies specific to acute ICP changes may not be generalizable to patients with chronic ICP elevation in outpatient settings. In this study, we investigated the effect of routine CSF drainage performed in the outpatient setting on ONSD.

## Methods

Our primary research question was to determine if the optic nerve sheath diameter demonstrates a measurable change via ultrasound assessment in real time for patients undergoing elective lumbar punctures correlated with changes in intracranial pressure. This study was approved by the University of Pittsburgh institutional review board (PRO14040248). Study investigators obtained written informed consent from all patients (or guardians of participants) in the study prior to enrollment.

We conducted a prospective cohort convenience study. The participants were patients of a collaborating neurologist at the University of Pittsburgh Medical Center who were scheduled to receive diagnostic lumbar puncture. These patients represented a unique sample for assessing the dynamics of acute ICP changes. All measurements took place during scheduled out-patient lumbar puncture clinics at the office of this neurologist. Study team members had specific training on how to perform ONSD measurements with POCUS. The PI (CS) has extensive training in POCUS, particularly in regard to ONSD measurements. He directly taught and supervised the other team members (comprised an EM POCUS fellow, EM residents and a medical student research assistant) for hands-on training across several sessions as well as their initial patient enrollments to ensure accuracy of technique and measurements. Subsequent, unsupervised scans were reviewed for accuracy of the saved measurements.

We included adult patients, ages 18–89, undergoing a non-emergent, scheduled lumbar puncture for diagnostic indications. We excluded pregnant women, and patients with prosthetic eyes, cataracts, glaucoma, or recent eye trauma or surgery. Patient who received their LP sitting up were excluded as CSF pressure measurement in the sitting position is not known to be reliable.

We calculated sample size based on prior studies of the relationship between ONSD and ICP in human subjects. ONSD measurement has a median inter-observer variation of ± 0.2–0.3 mm [19]. In an intrathecal infusion test conducted in human subjects that induced raised ICP in subjects, the ONSD-to-ICP ratio varied from 0.019 to 0.071 mm/cm [16]. Choosing conservative values of an ONSD-to-ICP ratio of 0.025 mm/cm, and an ICP change of 10 cm, we calculated that we would need to enroll 17 patients with elevated ICP. With the addition of a 35% buffer for dropout (i.e., inability to complete LP or done while sitting up), this yielded an anticipated enrollment of 23 patients. However, as the investigators were blinded to these data, all consecutive patients meeting inclusion criteria had these measurements obtained. We planned to enroll consecutive patients until at least 17 patients with elevated ICP had been included. Anticipating that 20% of the patients will have high intracranial pressure (> 25 cm), we determined that the anticipated number to include would be 115.

After screening, enrolling and obtaining written informed consent, the study investigators performed ultrasound (US) measurements of the participants’ ONSD. All study team members were competent in the use of US to acquire these views and accurately obtain the ONSD measurements. The investigators used a GE vivid i ultrasound machine with an 8–16 MHz high-frequency linear probe (GE Healthcare, Chicago, IL, USA). ONSD measurements were obtained with the patient supine to correspond with opening pressure measurements of patients in the lateral decubitus position. Each subject received four pre-LP US ONSD measurements: both eyes were evaluated in the traverse and sagittal planes. Consistent with previously published studies, the width of the ONSD was measured at 3 mm behind the globe, in the axis perpendicular to the optic nerve, with the patient supine [20]. These images were saved as digital files, de-identified from any patient information, locally to the US device itself for subsequent quality assurance review of the images and accuracy of measurement by the senior members of the research team. At this time, the patient was taken for their scheduled LP.

The second set of measurements was performed after the patients’ LP. The research team member, blinded from the patients pre- and post-LP pressures and amount of cerebral spinal fluid (CSF) removed, performed repeat measurements from the both of the participant’s ONSD in transverse and sagittal plane. These images were also saved locally to the device. After the images were saved, the neurologist performing the LP provided the participant’s opening pressure, closing pressure and amount of CSF he removed. These results were entered into our database as well as the ONSD measurements pre- and post-LP. Opening pressure > 25 cm was used to categorize patients as having increased ICP. However, we included all patients to assess the changes in ONSD pre- and post-LP in those both with and without elevated ICP in order to better understand the elasticity on the ONSD in vivo.

Patient data were de-identified and blinded to the research team members performing the ONSD measurements. However, demographic information of the patients was collected, including indication for LP (i.e., presently ongoing or resolved symptoms) and other existing co-morbidities.

We used descriptive statistics to characterize the study subjects. We calculated average ONSD for each eye as the arithmetic mean of sagittal and transverse measurements. For each eye, we compared sagittal and transverse and average ONSD before and after LP using two-tailed paired t test. We calculated change in ONSD at each eye and axis from pre-LP to post-LP. We also examined LP opening pressure in the subset of subjects with abnormal ONSD (> 5 mm). We used linear regression to measure association of sagittal, transverse, average, and change in ONSD with LP opening pressure, change in LP pressure, and volume of CSF drained. We considered p values < 0.05 to be significant. We used STATA 14.2 for all analyses (STATA Corp., College Park, Tx).

Data Availability: Anonymized data not published within this article will be made available by request from any qualified investigator.

## Results

**Fig. 1:**
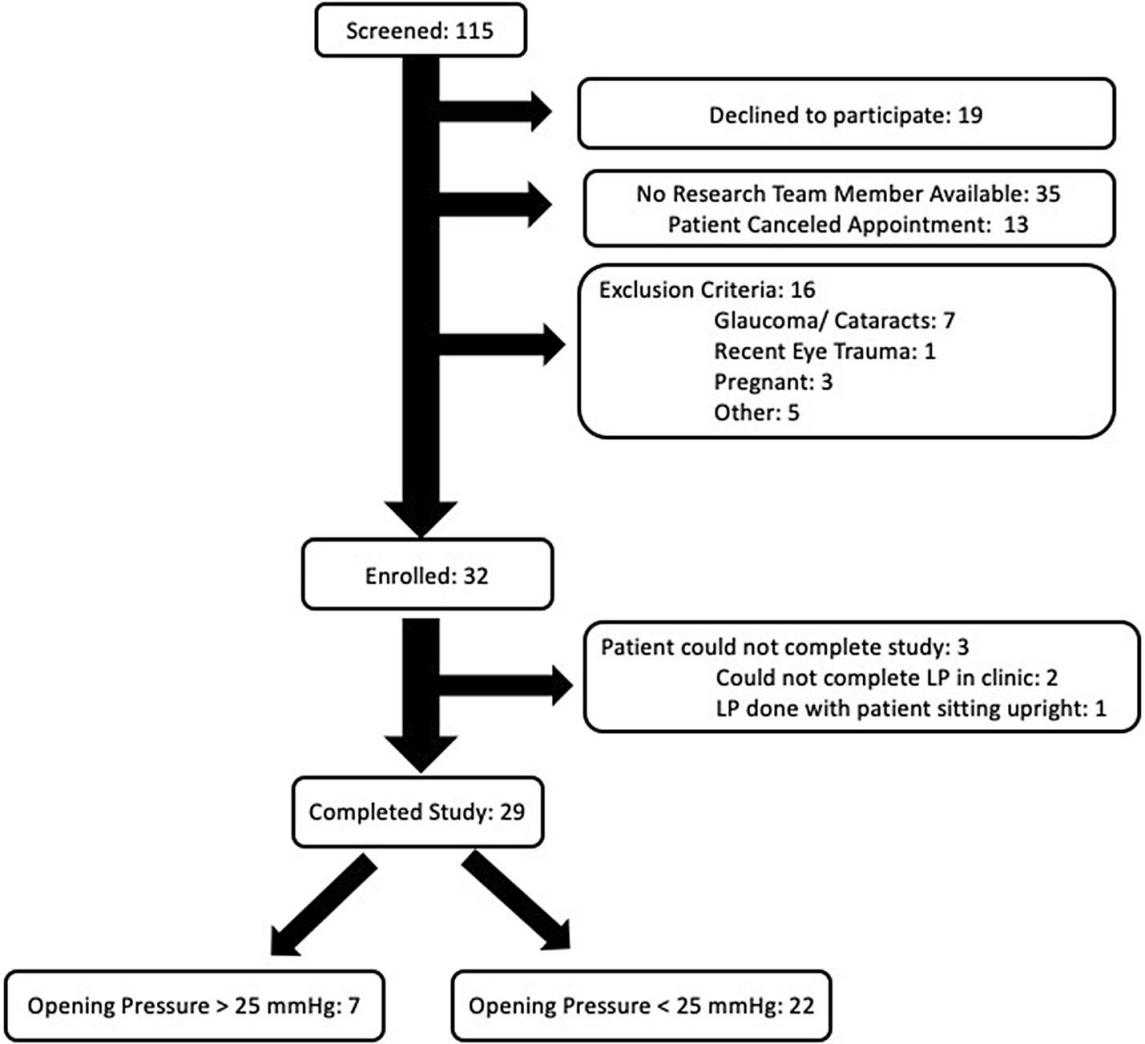
Patient enrollment flow chart. *LP* lumbar puncture

Between February 2015 and December 2017, we screened 113 patients. Thirty-two were enrolled with 29 completing all ONSD ultrasound and LP measurements (Fig. 1). We ended the study early due to slower than anticipated enrollment, which made the project no longer feasible.

The average patient age was 49.0 years (SD = 37–61, range 19–67 years of age) with 21 females (72.4%) and 26 (89.7%) white American (not Hispanic or Latino) with the remainder as Black or African American (Table 1). Twenty (69.0%) of the patients were scanned by investigator CS, 6 (20.7%) by DL, 2 (6.9%) by EB and 1 (3.4%) by MS. There was a mean of 68 min (SD = 48–88 min) between pre- and post-lumbar puncture ONSD measurements.

**Table 1:** Patient demographics

Characteristic	No (%)	Co-morbidities	No
Sex		Parkinson’s	1
Male	8 (27.59%)	Hypertension	5
Female	21 (72.41%)	Narcolepsy	1
Age	Avg = 49.03 (range 19–67)	Cervical stenosis	1
Race		Multiple sclerosis	2
White	26 (89.66)	Transverse myelitis	1
African America	3 (10.34%)	Depression/anxiety	2
		CAD	2
		Diabetes	5
Symptoms at present	No	Hyperlipidemia	2
Extremity paresthesias	6	Arthritis	1
Headache	3	Iron deficiency	1
Blurred vision	5	Lupus	1
Dizzy	3	Rheumatoid arthritis	1
Weakness (generalized)	4	Sjogren’s syndrome	1
Nausea	2	Reynaud’s	1
Back pain	1	Bipolar	3
		Pseudotumor cerebri	1
		Pre-eclampsia (history of)	1
		Psoriasis	1
		Hypothyroidism	1
		Prostate cancer	1
		Thyroid cancer	1

The skewness and kurtosis test of normality confirmed that continuous variables did not differ from normal except for LP opening pressure. Therefore, mean (SD) is used for most variables. The only continuous variable that deviated from normal distribution was LP opening pressure, where we report as median (IQR). For all participants, the median opening pressure was 19 cm (IQR 16–24) with average closing pressure of 13.5 cm with a difference of 6.9 cm (95% CI 3.9–10.0 cm). There was no statistically significant difference in opening pressures between the participants who had an ONSD measurement > 5 mm (average opening pressure = 21.3 cm) to those < 5 mm (20.2 cm) with a difference of 1.1 cm (*p* = 0.7395, 95% CI = − 8.0–5.7). The volume of CSF removal was normally distributed, with a mean of 13.4 cc’s (SD 4.2).

**Fig. 2:**
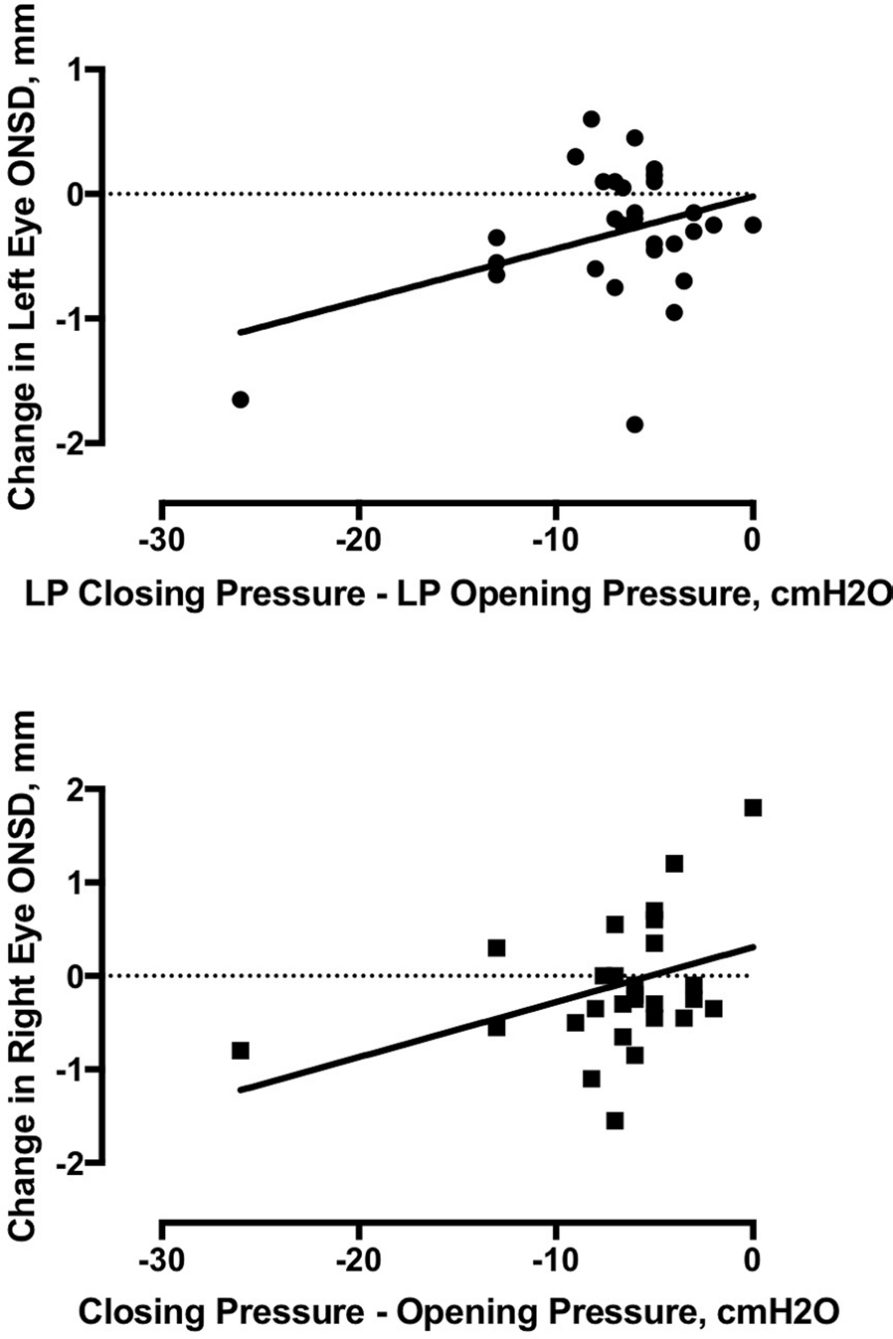
Linear regression analysis of change in between opening and closing pressures (in cmH_2_) compared against the change in optic nerve sheath diameter (mm), averaged between transverse and sagittal plan measurements, for the left and right eyes, respectively

We observed an increase of 0.3 mm in the left eye sagittal plane ONSD (*p* = 0.0318, 95% CI 0.03–0.6) as well as in the averaged left transverse and sagittal planes (delta = 0.3 mm, *p* = 0.0027, 95% CI = 0.1–0.5), but we did not observe any other reliable changes from pre- to post- lumbar puncture ONSD measurements (Table 2). We separately examined the 7 patients who had elevated ICP on opening pressure measurement (25 cm or greater; Table 3). Again, we observed an *increase* in the ONSD measurement for the left eye transverse plane (average increase of 0.8 mm, *p* = 0.0451, 95% CI = 0.03–1.1) and the averaged left eye measurements (average increase of 0.63 mm, *p* = 0.0293, 95% CI = 0.09–1.2), but no other changes from pre- to post-lumbar puncture ONSD measurements (Fig. 2; Tables 4, 5).

**Table 2:** Comparison of participant optic nerve sheath diameter, by plane of measurement, pre-lumbar puncture compared to post-lumbar puncture for all participants

	Average diameter (PRE)	Average diameter (POST)	Delta	*p*	95% CI
Left eye Transverse	5.09	4.8	0.29	0.0566	− 0.0088 to 0.5959
Left eye sagittal	5.02	4.72	0.3	0.0318	0.028 to 0.572
Left eye (averaged ONSD)	5.06	4.76	0.297	0.0037	0.1039 to 0.4896
Right eye transverse	4.84	4.86	− 0.02	0.8873	− 0.3451 to 0.2999
Right eye sagittal	5.04	4.82	0.223	0.0747	− 0.0237 to 0.4703
Right eye (averaged ONSD)	4.92	4.84	0.867	0.5042	− 0.1754 to 0.3488

**Table 3:** Comparison of participant optic nerve sheath diameter, by plane of measurement, pre-lumbar puncture compared to post-lumbar puncture, for participants with elevated intracranial pressure (opening pressure > 25 cm)

	Average diameter (PRE)	Average diameter (POST)	Delta	*p*	95% CI
Left eye Transverse	5.228571	4.4	0.8285714	0.0452	0.0245 to 1.2626
Left eye sagittal	5.257143	4.814286	0.442857	0.2336	− 0.3754 to 1.2612
Left eye (averaged ONSD)	5.242857	4.607143	0.6357142	0.0293	0.0894 to 1.1820
Right eye transverse	4.785714	4.8	− 0.0142856	0.9696	− 0.8939 to 0.8654
Right eye sagittal	5.4	5.016667	0.3833	0.2701	− 0.4115 to 1.1782
Right eye (averaged ONSD)	5.008333	4.891667	0.1166666	0.7165	− 0.6635 to 0.8968

**Table 4:** Linear regression analysis of ONSD measurements to opening pressure

Measurement	Correlation coefficient	*p*	*R*^2^	95% CI
Left eye transverse (Pre)	0.12	0.924	0.0003	− 2.502532 to 2.749281
Left eye sagittal (Pre)	0.28	0.801	0.0024	− 1.967434 to 2.524459
Left eye (averaged ONSD, pre)	0.24	0.848	0.0014	− 2.336629 to 2.82292
Right eye transverse (pre)	0.23	0.863	0.0011	− 2.442073 to 2.894984
Right eye sagittal (pre)	1.27	0.38	0.0286	− 1.652574 to 4.194005
Right eye (averaged ONSD, post)	0.797	0.589	0.011	− 2.194456 to 3.789119

**Table 5:** Linear regression analysis of ONSD measurements to volume of CSF removal

Measurement	Correlation coefficient	*p*	*R*^2^	95% CI
Right eye delta (transverse)	− 1.07	0.225	0.0502	− 2.824557 to .693879
Right eye delta sagittal	− 1.66	0.167	0.0669	− 4.057 to 0.7390722
Right eye delta (averaged)	− 1.64	0.146	0.0738	− 3.893995 to 0.6095693
Left eye delta (transverse)	1.18	0.209	0.0538	− 0.696745 to 3.04977
Left eye delta (sagittal)	0.96	0.358	0.0292	− 1.145068 to 3.072341
Left eye delta (averaged)	2.41	0.098	0.0915	− 0.4728556 to 5.283155

In linear regression models, there were no independent associations between the sagittal, transverse, average, and change in ONSD measurements with the observed LP opening pressure, change in LP pressure, and volume of CSF drained. Comparison of measurements in the 2 planes (transverse vs sagittal) at the same time period (pre- or post-lumbar puncture) demonstrates moderate to good levels of agreement (0.65–0.88; Table 6). However, there is a greater variation between eyes (left vs right) for measurements in the same plane and period of time (range = 0.33–0.84; Table 7).

**Table 6:** Agreement of measurements between planes (transverse and sagittal) for each eye, pre- and post-lumbar puncture

Measurement plane 1	Measurement plane 2	Cronbach’s alpha
L pre-transverse	L pre-sagittal	0.7629
R pre-transverse	R pre-sagittal	0.7485
L post-transverse	L post-sagittal	0.6456
R post-transverse	R post-sagittal	0.8825

**Table 7:** Agreement of measurements between eyes (right vs left) for each plane, pre- and post-lumbar puncture

Measurement Plane 1	Measurement plane 2	Cronbach’s alpha
Left eye pre, transverse	Right eye pre, transverse	0.3297
Left eye pre, sagittal	Right eye pre, sagittal	0.4405
Left eye post, transverse	Right eye post, transverse	0.8399
Left eye post, sagittal	Right eye post, sagittal	0.6885

## Discussion

This study provides evidence that the use of ONSD measurement via ultrasound (2D/B mode imaging with measurement of the ONSD 3 mm posterior to the globe in supine patients) does not correlate with real-time changes in ICP (as measured in cm of H_2_0) in ambulatory patients undergoing an elective lumbar puncture. Linear regression revealed no association between the sagittal, transverse, average, and change in ONSD measurements with the observed LP opening pressure, change in LP pressure, or volume of CSF drained, except for an *increase* in the ONSD measurement for the left eye transverse plane (average increase of 0.8 mm, *p* = 0.0451, 95% CI = 0.03–1.1) and the averaged left eye measurements (average increase of 0.63 mm, *p* = 0.0293, 95% CI = 0.09–1.2).

Our results suggest that clinically significant changes in ONSD measurements via ocular ultrasound may not be seen among ambulatory patients. Only ONSD in the left transverse and left averaged (transverse and sagittal) planes changed before and after LP. The direction of this change was actually an increase in the ONSD, contrary to our hypothesis. Moreover, the magnitude of this change (0.3 mm) is not clinically significant, being much less than the standard deviation of observed ONSD measurements and potentially less than inter-rater and intra-rater variation in measurement. These findings were no different when we separated out the patients with proven elevated ICP.

There was moderate to good correlation between the measured planes (transverse vs sagittal) for the ONSD at the same periods of time (pre-LP vs post-LP). However, there was a wide range of agreement in measurements between left and right eyes (range of agreement 0.33–0.84). This finding suggests an agreement in the ONSD measurement between planes of the same eye, but wider range of agreements between eyes (i.e., asymmetric ONSD among this patient population). This could be due to the underlying etiology of the ONSD enlargement, as many of the patients were presenting for neurologic complaints with workups for multiple sclerosis. Therefore, one explanation may be that the enlargement in a unilateral eye could be due to inflammation (e.g., optic neuritis), as opposed to increased intracranial pressure, and may not demonstrate changes with intracranial pressures. No ONSD measurement in either plane, eye or time period (pre-LP or post-LP) demonstrated a significant relationship with opening intracranial pressure or amount of CSF removed.

A recent publication by Wang et al. reported a higher correlation between ONSD and changes in lumbar puncture (*r* = 0.724, 95% CI 0.470–0.876; *p* < 0.001) among patients admitted for suspected elevated ICP [21]. However, their reported statistical correlation may be limited in clinical application. Their results show that a patient with a 20-cm drop in ICP may have a measured ONSD change of as little as 0.5 mm on bedside ultrasound. This raises the question of whether a potential observed change of 0.5 mm is clinically useful or detectable, particularly with previously published inter-rater variability of 0.2–0.3 mm [19]. Furthermore, the validity of these results has been questions, as noted in a letter to the editor addressing this particular publication [22]. Therefore, though studies like this and others support a statistical correlation between changes in ICP and ONSD measurements, it is important to keep in mind potential the clinical limitations and applicability of this measurement when attempting to apply to patient care.

A recent systematic review and meta-analysis of 71 studies, inclusive of adult and pediatric populations, report pooled sensitivity, specificity, positive likelihood ratio, and negative likelihood ratio of ONSD measurements to be 97% (95% CI 92–99%), 86% (CI 74–93%), 6.93 (CI 3.55–13.54), and 0.04 (CI 0.02–0.10), respectively [10]. However, in contrast to our study, the included papers used in their meta-analysis predominantly come from a patient population with known intracranial pathology (18 with traumatic brain injury, 26 without and 27 mixed). This is in contrast to our study, examining ambulatory patients without known elevations in ICP. We may not have observed as noticeable results with potential etiologies for enlarged ONSD measurements from etiologies other than elevation in ICP. The contrast in patient populations suggests that this method to assess for elevation in ICP via ONSD may not be generalizable to all patient populations.

## Limitations

The study has several limitations. First, the study was stopped early secondary to slow enrollment. Patients did not consent to participate in the study at a rate we felt was feasible for continued data collection. Though we did not reach our anticipated target enrollment, there were enough patients to detect some differences between left eye (transverse, averaged transverse and sagittal) ONSD measures pre- and post-LP, and to provide point estimates for the changes in ONSD. The study is also limited in that we used a convenience sample population of ambulatory patients undergoing scheduled lumbar punctures. The majority of this population were white females, potentially limiting generalizability. Moreover, this patient population may have had changes in ONSD from other etiologies, such as inflammation from underlying neurologic condition, opposed to elevated intracranial pressures. Since we used a convenience sample of patients during their scheduled LP, we were only able to record pre- to post- LP within the timeframe of their office visit. We cannot exclude potential changes in ONSD that occur longer after LP.

## Conclusions

Our study suggests that there are limitations to the use and application of ONSD ultrasound measurements in ambulatory patients, in contrast to prior studies in critically ill patients [1–3, 7–9, 11, 23] In this study, we were not able to consistently detect changes in the ONSD across each eye or multiple planes of view. Observed changes in single eyes were too small in magnitude to trust for clinical decision making. We did not observe any correlation between ONSD measurements by ultrasound and opening pressures, change in pressure or amount of CSF removed, either in all patients or in the group of patients with elevated ICP. In addition, we did not find a relationship between enlarged ONSD and ICP.

While point-of-care ultrasound has many applications for bedside assessments and clinical decision making, this study suggests caution in using ultrasound measurements of the ONSD in ambulatory patients.

## Data Availability

The datasets used and/or analyzed during the current study are available from the corresponding author on reasonable request

## Abbreviations

ONSD: Optic nerve sheath diameter
LP: Lumbar puncture
CSF: Cerebral spinal fluid
ICP: Intracranial pressure
POCUS: Point-of-care ultrasound

## References

[1] Gangemi M, Cennamo G, Maiuri F, D’Andrea F (1987). Echographic measurement of the optic nerve in patients with intracranial hypertension. Neurochirurgia (Stuttg)..

[2] Galetta S, Byrne SF, Smith JL (1989). Echographic correlation of optic nerve sheath size and cerebrospinal fluid pressure. J Clin Neuroophthalmol..

[3] Robba C, Santori G, Czosnyka M (2018). Optic nerve sheath diameter measured sonographically as non-invasive estimator of intracranial pressure: a systematic review and meta-analysis. Intensive Care Med.

[4] De Moraes CG (2013). Anatomy of the visual pathways. J Glaucoma.

[5] Selhorst JB, Chen Y (2009). The optic nerve. Semin Neurol.

[6] Moretti R, Pizzi B, Cassini F, Vivaldi N (2009). Reliability of optic nerve ultrasound for the evaluation of patients with spontaneous intracranial hemorrhage. Neurocrit Care.

[7] Liu D, Li Z, Zhang X (2017). Assessment of intracranial pressure with ultrasonographic retrobulbar optic nerve sheath diameter measurement. BMC Neurol..

[8] Jeon JP, Lee SU, Kim S-E (2017). Correlation of optic nerve sheath diameter with directly measured intracranial pressure in Korean adults using bedside ultrasonography. PLoS ONE.

[9] Raffiz M, Abdullah JM (2017). Optic nerve sheath diameter measurement: a means of detecting raised ICP in adult traumatic and non-traumatic neurosurgical patients. Am J Emerg Med.

[10] Koziarz A, Sne N, Kegel F (2019). Bedside optic nerve ultrasonography for diagnosing increased intracranial pressure. Ann Intern Med.

[11] Dubourg J, Javouhey E, Geeraerts T, Messerer M, Kassai B (2011). Ultrasonography of optic nerve sheath diameter for detection of raised intracranial pressure: a systematic review and meta-analysis. Intensive Care Med.

[12] Ohle R, McIsaac SM, Woo MY, Perry JJ (2015). Sonography of the optic nerve sheath diameter for detection of raised intracranial pressure compared to computed tomography: a systematic review and meta-analysis. J Ultrasound Med.

[13] Williams P (2017). Optic nerve sheath diameter as a bedside assessment for elevated intracranial pressure. Case Reports Crit Care..

[14] Hassen GW, Al-Juboori M, Koppel B, Akfirat G, Kalantari H (2018). Real time optic nerve sheath diameter measurement during lumbar puncture. Am J Emerg Med.

[15] Maissan IM, Dirven PJAC, Haitsma IK, Hoeks SE, Gommers D, Stolker RJ (2015). Ultrasonographic measured optic nerve sheath diameter as an accurate and quick monitor for changes in intracranial pressure. J Neurosurg.

[16] Hansen HC, Helmke K (1997). Validation of the optic nerve sheath response to changing cerebrospinal fluid pressure: ultrasound findings during intrathecal infusion tests. J Neurosurg.

[17] Bäuerle J, Niesen WD, Egger K, Buttler KJ, Reinhard M (2016). Enlarged optic nerve sheath in aneurysmal subarachnoid hemorrhage despite normal intracranial pressure. J Neuroimaging.

[18] De Masi R, Orlando S, Conte A (2016). Transbulbar B-mode sonography in multiple sclerosis: clinical and biological relevance. Ultrasound Med Biol.

[19] Ballantyne S, O’Neill G, Hamilton R, Hollman A (2002). Observer variation in the sonographic measurement of optic nerve sheath diameter in normal adults. Eur J Ultrasound..

[20] Soldatos T, Chatzimichail K, Papathanasiou M, Gouliamos A (2009). Optic nerve sonography: a new window for the non-invasive evaluation of intracranial pressure in brain injury. Emerg Med J..

[21] Wang L, Chen L, Chen Y (2018). Correlation between noninvasive ultrasonography and dynamically monitored intracranial pressure. JAMA Ophthalmol..

[22] Hoffman RS (2018). Measuring optic nerve sheath diameter as a proxy for intracranial pressure. JAMA Ophthalmol..

[23] Moretti R, Pizzi B (2011). Ultrasonography of the optic nerve in neurocritically ill patients. Acta Anaesthesiol Scand.

